# Host-specific monogeneans parasitizing freshwater fish: The ecology and evolution of host-parasite associations

**DOI:** 10.1051/parasite/2024058

**Published:** 2024-10-03

**Authors:** Andrea Šimková

**Affiliations:** Department of Botany and Zoology, Faculty of Science, Masaryk University Kotlářská 2 611 37 Brno Czech Republic

**Keywords:** Fish parasites, Monogeneans, Host specificity, Species coexistence, Host-parasite coevolution, Genetic coadaptation

## Abstract

Monogenea (Platyhelminthes), mainly gill and fin ectoparasites of fish, are often recognized as host specific and morphologically and ecologically diverse. These parasites exhibit high species diversity at the level of host species or individual fish specimens. Using case studies, especially those widely performed in *Dactylogyrus* parasitizing cyprinoid fish, this article presents current knowledge on the ecology and evolution of congeneric gill monogeneans. The important aspects of the ecology of congeneric monogeneans are highlighted, in particular: host specificity expressed at several host levels (from strict specificity to phylogenetic specificity), microhabitat specificity expressed by restricted positions on fish gills to facilitate intraspecific mating, and the link between microhabitat preference and morphological adaptation (i.e., sclerotized structures of the haptor) or reproductive isolation. From the evolutionary perspective, this study focused on the processes of the speciation and diversification of congeneric monogeneans, highlighting the role of host switch as the most prominent coevolutionary event, accompanied in some cases mostly by intrahost speciation or cospeciation, as revealed by cophylogenetic studies. Here, important knowledge on evolutionary patterns of host specificity, microhabitat specificity, and morphological adaptation is presented. Host-specific monogeneans may represent an important tool for studying the historical biogeography of their hosts. Specifically, in the case of freshwater fish hosts exhibiting disjunctive distribution, they reflect both historical and contemporary contacts. The role of host-specific congeneric monogeneans in revealing historical intercontinental and intracontinental contacts between freshwater fish is highlighted. Finally, the importance of the role of genetic coadaptation, limiting the presence of host-specific monogeneans in hybrid fish, is emphasized.

## Abstract (fr)

Les Monogènes (Plathelminthes), principalement ectoparasites des branchies et des nageoires des poissons, sont souvent reconnus comme spécifiques à leur hôte et diversifiés morphologiquement et écologiquement. Ces parasites présentent une grande diversité d’espèces au niveau des espèces hôtes ou des spécimens individuels de poissons. À l’aide d’études de cas (en particulier celles largement réalisées chez les *Dactylogyrus* parasitant les poissons cyprinoïdes), les connaissances actuelles sur l’écologie et l’évolution des monogènes branchiaux congénères sont présentées. Les aspects importants de l’écologie des monogènes congénères sont mis en évidence, en particulier la spécificité à l’hôte exprimée à plusieurs niveaux de l’hôte (de la spécificité stricte à la spécificité phylogénétique), la spécificité du microhabitat exprimée par des positions restreintes sur les branchies des poissons pour faciliter l’accouplement intraspécifique, et le lien entre la préférence du microhabitat et l’adaptation morphologique (c’est-à-dire les structures sclérifiées du hapteur) ou l’isolement reproductif. Du point de vue évolutif, l’étude a été concentrée sur les processus de spéciation et de diversification des monogènes congénères, soulignant le rôle du changement d’hôte comme l’événement coévolutif le plus important, accompagné surtout dans certains cas de spéciation ou de cospéciation intra-hôte, comme le révèlent les études cophylogénétiques. Des connaissances importantes sont présentées ici sur les modèles évolutifs de spécificité d’hôte, de spécificité de microhabitat et d’adaptation morphologique. Les monogènes spécifiques à l’hôte peuvent représenter un outil important pour étudier la biogéographie historique de leurs hôtes. Plus précisément, dans le cas des poissons d’eau douce hôtes présentant une distribution disjonctive, ils reflètent à la fois des contacts historiques et contemporains. Le rôle des monogènes congénères spécifiques à leur hôte dans la révélation des contacts intercontinentaux et intracontinentaux historiques entre poissons d’eau douce est mis en évidence. Enfin, l’importance du rôle de la coadaptation génétique limitant la présence de monogènes spécifiques à l’hôte chez les poissons hybrides est soulignée.

## Introduction: diversity of organisms parasitizing fish

In the context of more complex life forms (i.e., excluding viruses and bacteria), fish parasites exhibit huge diversity in terms of lineages, species, or genetic variants. They include various protozoan taxa, many of them having serious economic consequences in aquaculture conditions (e.g. [8, 70]), and high taxonomic variety of metazoan parasites, exhibiting an enormous range of morphological, molecular, and ecological adaptations. Such metazoan parasites include Myxozoa, Platyhelminthes (Monogenea, Trematoda, and Cestoda), Nematoda, Acanthocephala, Arthropoda (Crustacea, and rare Acarina), Hirudinea, and Mollusca. Some of them even exhibit a sophisticated capacity for behavioral manipulation of their fish hosts, mostly documented for the larval stages of trematodes and cestodes, in order to facilitate their transmission to definitive hosts (e.g., [21, 38]). The majority of the above-mentioned metazoan parasite groups are species-rich and have been well documented by frequent parasitological surveys of fish. There are also some fascinating lesser-known cases of fish parasitism, such as freshwater mussels and European bitterling (*Rhodeus sericeus*); here, each of the interacting partners may play the role of host or parasite depending on the specific stage of their life cycle [46].

## Monogeneans – parasites with unique life history

Among fish parasites, monogeneans, predominantly representing fish ectoparasites living on the gills, skin, and fins, express unique biological traits that make them a suitable biological object for ecological and evolutionary studies (including host-parasite coevolution). With respect to oviparous fish monogeneans, their life cycle includes a free-living larval stage, or oncomiracidium, which actively searches for a host. After reaching the host, it attaches itself to the body surface, migrates to its final destination (the gills), and moves towards specific microhabitats, e.g., a preferred position on the gills where reproduction of adult parasites takes place. However, fish monogeneans also include viviparous representatives (gyrodactylids), and even some rare endoparasitic genera with restricted species diversity (e.g., *Enterogyrus*, *Urogyrus*, and *Acolpenteron*), for which life cycles have not yet been fully clarified. Monogeneans exhibit high species diversity as well as high morphological variability, often recognized even within a single genus (see, for example, Pugachev et al. [43] for a variety of central hooks (termed anchors), marginal hooks, dorsal connective bars, and ventral connective bars in the highly diversified monogenean genus *Dactylogyrus*). Such sclerotized parts of the attachment organ (haptor) have been considered the main morphological characters for taxonomical identification, and, from an evolutionary point of view, reflect morphological adaptation to the associated host species.

In fact, monogeneans are usually considered to be highly host-specific. However, when considering the host specificity of a given parasite species, several criteria should be carefully considered. Poulin et al. [41] highlighted different perspectives on host specificity that should be considered when studying monogenean host specificity. First, host specificity has generally been expressed by the simple number of host species infected by a given parasite species, which is termed basic host specificity, and thus a parasite infecting a single host species is considered a strict specialist (see also Šimková et al. [61] for delimitation of the host specificity of congeneric monogeneans (*Dactylogyrus* spp.)). However, for a parasite species infecting more than one host species, the level of parasite infection, usually expressed by quantitative data such as prevalence, abundance, and intensity of infection (see Bush et al. [10]), should also be considered when expressing host specificity, because although one parasite species may infect different host species, it may express a high abundance only on one of them, leaving the other host species less parasitized. In contrast, another parasite species infecting the same number of host species (i.e., having the same basic host specificity) may express similar abundance on all examined host species. In such a hypothetical case, the first parasite species is more host-specific when compared to the second one. The use of quantitative data on the calculation of host specificity was proposed and applied to fish ectoparasites, for example, by Rohde [49] or Rohde & Rohde [53]. However, a real evaluation of host specificity for large assemblages of congeneric monogenean species is often limited to published records, and quantitative infection-related data sets pertaining to monogeneans infecting highly diversified fish lineages are rarely at the researcher’s disposal.

Expression of the host specificity of a given parasite species should also consider the phylogenetic relationships between host species. For example, one parasite species may infect different host species, all of them being members of the same clade, while a second parasite species may infect the same number of host species, although its hosts each belong to different phylogenetic lineages (however, both parasite species express the same level of basic host specificity). In such a case, the first parasite species is more specific than the second. Formerly, Poulin & Mouillot [42] proposed an index that considers the taxonomical difference between host species, i.e., the number of taxonomic steps required to reach the common ancestor of both species. The era of molecular phylogenies also opened possibilities to incorporate host phylogenies into the expression of the phylogenetic host specificity of a parasite species. The concept of phylogenetic host specificity based on simple semiquantitative indexes has been applied in several studies investigating the evolution and determinants of the host specificity of monogeneans [14, 29, 33, 61]. For example, Šimková et al. [61] classified *Dactylogyrus* species parasitizing cyprinoids with European distribution into: (1) strict specialists living on a single host species, (2) intermediate specialists living on two or more congeneric host species, (3) intermediate generalists living on non-congeneric hosts belonging to the same clade, (4) generalists living on different host species, however still the members of one taxonomical unit (host subfamily), and (5) real generalists living on different host species from different taxonomical units (different subfamilies in the case of Šimková et al. [61]). The host specificity of monogeneans is also correlated with host sample size and parasite phylogeny [29, 61]; however, other attributes such as the morphology and ecology of both parasites and hosts have been associated with host specificity [33, 61]. Thus, high host specificity may be an artefact of inadequate sampling [40]. Congeneric monogenean species often express narrower host specificity at the local level of study than at the regional level; however, it was reported that a single host species found for a given monogenean parasite species at the local level of investigation is a common (i.e., the most frequent) host for this monogenean parasite species at a wider geographical level, i.e., regional level of investigation, and such a host species is therefore important for sustaining the parasite population, whereas an additional (less used) host alone is not sufficient for monogenean specialist maintenance [61].

The mechanism proposed for the evolution of host specificity in parasites by Kawecki [26] is based on the prediction that an initially generalist parasite evolves toward specific lineages, each selecting one host species, and then the parasite evolves a host preference; thus, a specialist can coevolve faster in response to host evolution (i.e., defense mechanisms) than a generalist parasite. Specialization, which is closely related to host specificity, was formerly proposed as an evolutionary ‘dead end’ [69], with specialist lineages unlikely to evolve into generalist lineages. However, some host-parasite studies previously suggested the opposite trend, i.e., generalists evolved from specialists (e.g. [25, 54]), or others proposed that some generalists specialize on a particular host species, i.e., they are resource specialists, but retain their ability to become generalists under specific environmental conditions [36, 37].

The phylogenetic reconstructions of congeneric monogeneans suggest that narrow host specificity (a strict or intermediate level of host specificity) is an ancestral character state and that different degrees of wider host specificity represent derived conditions. Transition from specialists to generalists appeared in phylogenetic trees of congeneric monogeneans multiple times. At the same time, phylogenetically related monogenean parasite species at terminal positions on the phylogenetic tree (i.e., the individual species belonging to a single monophyletic group) develop either specialist or generalist behavior [14, 33, 61].

## Ecology of congeneric monogeneans

Congeneric monogeneans often tend to coexist on the same host species in high species numbers. High monogenean species diversity was documented at the level of host species and even at the level of individual fish [57, 58]. Congeneric monogenean species of such highly diverse communities, typically species living at low density, exhibit, however, a strong tendency to be aggregated, and show microhabitat preference, i.e., parasite species infecting the fish gills select a specific microhabitat basically to increase the chances of mating [47, 59].

**Figure 1:**
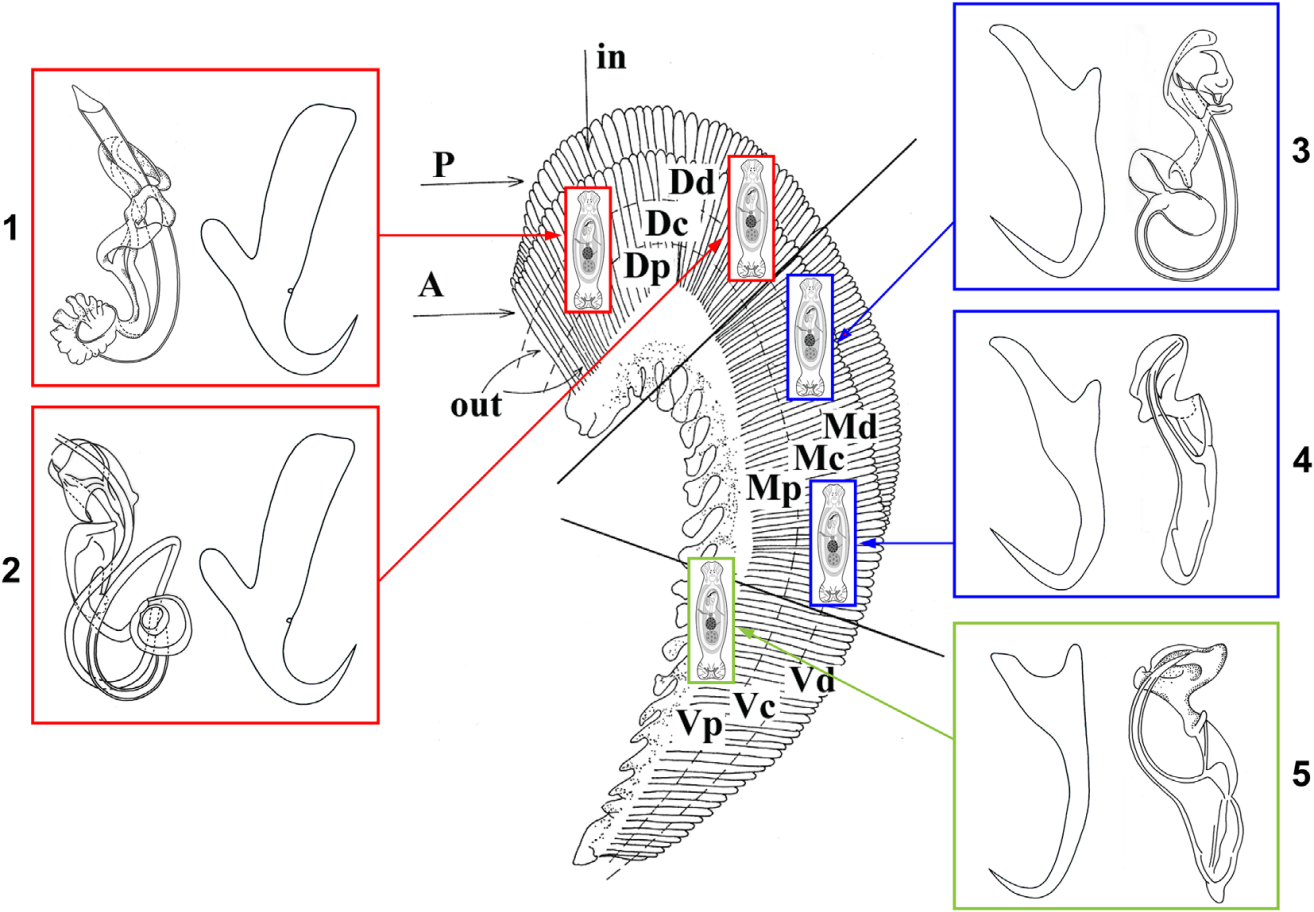
Coexistence of five *Dactylogyrus* spp. on a single gill arch. *Dactylogyrus* sp. 1 and *Dactylogyrus* sp. 2 (in red) exhibit the same anchor morphology (the sclerotized parts of the haptor) and similar microhabitat positions (the dorsal segment (D) and central area of a gill arch (c), but differ in the shape and size of their copulatory organ. *Dactylogyrus* sp. 3 and *Dactylogyrus* sp. 4 (in blue) exhibit the same anchor morphology and similar microhabitat positions (the medial segment (M) and central area of a gill arch (c), but differ in the shape and size of their copulatory organ. *Dactylogyrus* sp. 5 (in green) has a different anchor morphology when compared to *Dactylogyrus* sp. 1–sp. 4, and is positioned in the ventral segment (V) and proximal area (p) of a gill arch.

**Figure 2:**
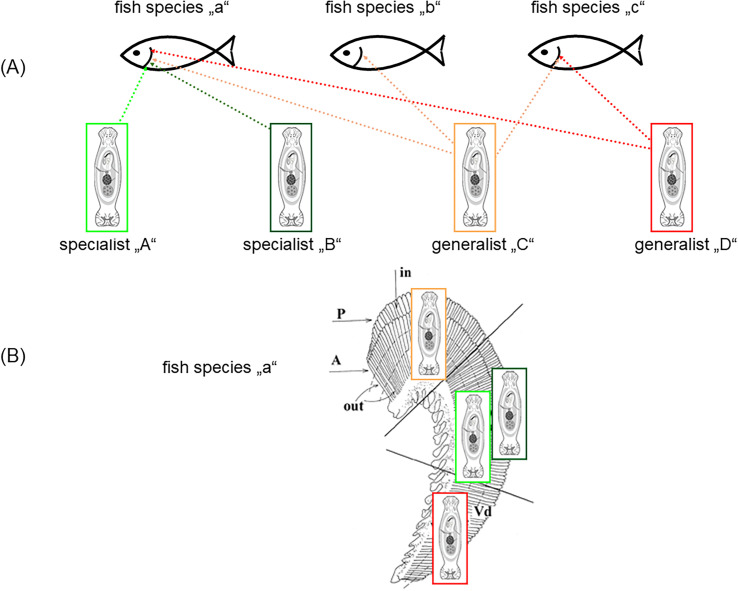
Microhabitat segregation on a gill arch (B) in respect to host specificity (A). (A) Congeneric monogenean species “A” and “B” are host-specific for a fish species “a”. Congeneric monogenean species “C” and “D” are generalists with different host ranges, i.e., species C infects three fish species (“a”, “b” and “c”) and species D infects two fish species (“a” and “c”). (B) Microhabitat position of four congeneric monogenean species parasitizing a fish species “a” on the gill arch. While two host-specific monogenean species (in light and dark green) for a fish “a” occupied closely related microhabitat positions within fish species “a”, two generalist species (in orange and red) infecting a wide range of fish species occupied distant microhabitat positions on the gill arch of fish species “a”.

Rohde [48, 50, 51] proposed that interspecific competition does not play a significant role in fish ectoparasite communities. Several mechanisms facilitating the coexistence of monogenean species on the same host have been proposed. Applying the aggregation model of coexistence, high intraspecific aggregation exceeding interspecific aggregation was found as a support for the coexistence of congeneric monogenean species [57]. Šimková et al. [59] showed that the morphological similarity in the attachment organ (haptor) expressed by the morphometry of sclerotized structures increases with niche overlap, which means that congeneric species, here *Dactylogyrus* species, positioned in the same or closely-located microhabitats express similar haptor morphology (Fig. 1). This also supports the niche specialization hypothesis, i.e., species that colonize the same niche exhibit similarities in organs involved in resource exploitation – here, the attachment organ of congeneric monogeneans. Microhabitat selection and its output – the preferred microhabitat positions in congeneric monogenean species – should also preclude interspecific hybridization (reinforcement of reproductive barriers). However, if congeneric species exhibiting similar haptor morphologies occupy the same or closely-located microhabitats, they then differ in the shape or size of their copulatory organ, which reinforces their reproductive isolation [50, 52, 59]. Microhabitat segregation in respect to host specificity was documented among congeneric monogenean species [59]. While two specialists both infecting the same host species tend to occupy closely located microhabitat positions within the gills of this host, two generalist species, each infecting a wide range of host species, tend to occupy the most distant microhabitat positions when living with congeneric specialist parasites on the same host [59, 62] (see Fig. 2). Thus, specialist adaptations also seem to facilitate the coexistence of congeneric monogenean species.

## Evolution of congeneric monogeneans in the context of fish-monogenean coevolution

Considering the uniqueness of monogenean life traits, close fish host-monogenean parasite co-evolutionary associations are expected. Formerly, a link between parasite speciation and host specificity was proposed [7], and cospeciation was therefore hypothesized for host-specific parasites. During cospeciation, host lineages speciate, and parasites also speciate on the descendant host species, which is usually illustrated by congruent host and parasite phylogenies. Because monogeneans are parasites with a direct life cycle and high host specificity, they are expected to cospeciate with their hosts [15, 39]. Therefore, former coevolutionary studies using fish-monogenean systems addressed questions about the link between host specificity and cospeciation [15, 60]. However, Desdevises et al. [15], focusing on *Lamellodiscus* (Monopisthocotylea), a group of gill monogenean parasites specific to marine fish of the Sparidae, showed rapid speciation by host switch for *Lamellodiscus* on their fish hosts living in sympatry, but did not reveal the roles of cospeciation and intrahost speciation (a case of sympatric speciation in parasites – see also Šimková et al. [60]). Host switch is a common coevolutionary scenario resulting in incongruent host-parasite phylogenies; however, other coevolutionary events, i.e., intra-host speciation (parasite duplication), failure to diverge, and sorting events (also termed as lineage sorting or loss), may also generate incongruent phylogenies for hosts and their parasites. Coevolutionary studies have since widely documented incongruent fish-monogenean phylogenies [23, 32, 44, 55, 60, 63, 72]. However, the incongruence of host and parasite phylogenetic trees with respect to host switch must be interpreted with caution, as, in the case of phylogenetically close host species, multiple host switches followed by parasite speciation may even result in similar topologies of the phylogenetic trees of hosts and parasites [12, 17, 18].

Cophylogenetic reconstructions performed for fish-monogenean systems in various geographical regions have constantly shown that host switch plays an important role in the evolutionary history of congeneric monogeneans, likely resulting from the sympatric distribution of their hosts (as supported e.g., in *Dactylogyrus* parasitizing endemic cyprinoids in southern Europe [4], in *Sciadicleithrum* and *Gussevia* parasitizing Neotropical cichlids [55], and in *Cichlidogyrus* parasitizing Lake Tanganyika cichlids [44]). This is often explained by the high diversification of phylogenetically-related fish species facilitating the host switching of specific parasites often among diverse congeneric fish living in sympatry, such as in the case of cyprinoids in the Mediterranean area [3]. Benovics et al. [3] proposed that host switch is the primary speciation event in *Dactylogyrus*, followed by intrahost speciation only if host switching is not possible due to geographical isolation or phylogenetic divergence among fish species living in sympatry. However, some studies have also highlighted the role of intrahost speciation acting together with host switch as the frequent coevolutionary events generating congeneric monogenean diversity in several fish host groups, e.g., *Dactylogyrus* parasitizing central European cyprinoids, mostly including leuciscids [60], *Cichlidogyrus* parasitizing West African cichlids [32], and *Thaparocleidus* in Asian pangasiid catfishes [63]. All these studies strongly support the idea that the high host specificity of monogeneans is not linked to cospeciation. The intrahost speciation of congeneric monogeneans raises questions on the evolution of microhabitat selection, which can be investigated through preferred niches [61]. Following Rohde [51], niche segregation is closely related to reproductive isolation between congeneric parasite species on/in the same host species to prevent competition and to increase intraspecies mating contacts [34, 57]. Šimková et al. [61], applying the mapping of preferred niche position using three niche dimensions (gill arch, gill segment, and gill area), showed that congeneric monogeneans speciating within one host (i.e., the output of a single intrahost speciation) tend to occupy niches differing at least in one niche dimension.

The study of host-parasite coevolution using specific fish-congeneric monogenean systems, such as in the case of *Dactylogyrus*-Barbinae (cyprinoid fish mostly from the *Luciobarbus* and *Barbus* genera widely distributed in the peri-Mediterranean) inferred that host-parasite cospeciation is frequent in phylogenetically-divergent host lineages; however, host switch still plays the principal role in the speciation of *Dactylogyrus*, allowing some *Dactylogyrus* species to parasitize a wide range of congeneric hosts [4]. The underlying mechanisms triggering speciation in viviparous *Gyrodactylus* displaying phylogenetic host specificity to goby hosts (belonging to the *Pomatoschistus* genus) were studied by Huyse & Volckaert [24]. Their study showed that host-specific gill *Gyrodactylus* of gobies originated from host switch from non-goby fish, and consequently that *Gyrodactylus* among goby hosts speciated by host-switching events, while less specific fin *Gyrodactylus* resulted from cospeciation in several host-associated species complexes. Thus, Huyse & Volckaert [24] highlighted that phylogenetically conserved host-switching may mimic the phylogenetic signature of cospeciation.

Benovics et al. [6] investigated the coevolutionary events shaping intra-species diversification by focusing on two generalist *Dactylogyrus* species; however, these species still differed in host specificity. While host-parasite cospeciation was shown to play an important role in diversification within *D. folkmanovae*, a parasite expressing a unique genetic variant in each host population (i.e., being more specific), diversification within *D. vistulae*, a parasite exhibiting identical genetic variants in multiple populations (i.e., being a real generalist), was found to be driven mainly by host switching. However, to generalize the pattern of intra-species diversification in *Dactylogyrus* species or even in congeneric monogeneans in respect to their host specificity, the various congeneric monogenean species should be studied in the future.

Concerning host switch, even host-specific monogeneans primarily associated with a given fish group/taxon may sometimes secondarily colonize, speciate, and adapt to hosts originally not associated with these monogeneans. Phylogenetic reconstructions of *Dactylogyrus* spp. associated with cyprinoids showed host switch and the consequent speciation of *Dactylogyrus* in Catostomidae in North America (currently 9 species, according to Kuchta et al. [29], see Šimková et al. [67]) and in Percidae in Eurasia (2 species, see Šimková et al. [60]).

## Host-specific monogeneans reflecting the historical biogeography of their freshwater fish hosts

Parasites exhibiting close coevolutionary associations with their hosts may represent a useful tool for inferring the historical biogeography of the hosts, especially in the case of hosts with disjunctive or fragmented distribution. Host-specific monogeneans are ideal candidates to shed light on the biogeographical history and/or more contemporary contacts of their freshwater fish hosts historically associated with a once contiguous landmass or some paleogeographical event.

Host-specific *Dactylogyrus* were used to infer the biogeographical routes of their cyprinoid hosts in the peri-Mediterranean, specifically in the Balkans [1, 4], the Apennine Peninsula [4], the Iberian Peninsula [2, 4] and Northwest Africa [4, 65]; all these regions are characterized by very high endemism of freshwater fish fauna. First, Benovics et al. [1] showed that the diversification of *Dactylogyrus* in the Balkans is associated with the historical dispersion of their cyprinoid hosts; however, it also reflects the more recent human‐induced introduction of non‐native cyprinoid species into the Balkans and Apennines, and contacts between non-native and endemic cyprinoids. In addition, their study revealed that endemic cyprinid species harbored *Dactylogyrus* species of different origins, this probably resulting from multiple host switching.

Later, Šimková et al. [65] performed phylogenetic analyses using host-specific *Dactylogyrus* spp. including the species endemic to the Iberian Peninsula and species endemic to North Africa. They confirmed the independent historical dispersion of cyprinoids from Asia (or Eurasia) to North Africa, which was previously suggested by the molecular phylogenies of cyprinoids [71, 73], and revealed multiple historical contacts between Iberian and North African cyprinoids associated with at least two host switches of *Dactylogyrus*, followed by the subsequent speciation and diversification of these monogeneans in both Iberia and North Africa. This particularly concerns cyprinoids of the Cyprinidae that are naturally distributed mostly in the southern parts of Eurasia and throughout the whole of Africa. Specifically, one dispersal event for cyprinids of the Torini (*Carasobarbus* species and *Pterocapoeta maroccana*) and another for cyprinids of the Barbini (*Luciobarbus* species) were inferred from the molecular phylogeny of host-specific *Dactylogyrus* [65]. They showed that *Dactylogyrus* spp. from *Carasobarbus* spp. originated from Asian cyprinids, which may be explained by the historical Gomphotherium land bridge between Africa and Asia in the Middle Miocene, while *Dactylogyrus* spp. from North African *Luciobarbus* spp. originated from European cyprinids, indicating the Northern route of historical *Dactylogyrus* spp. dispersion to Northwest African *Luciobarbus* species. For Iberian *Dactylogyrus* species parasitizing cyprinids, specifically *Luciobarbus* spp., Šimková et al. [65] showed that one Iberian *Dactylogyrus* lineage was phylogenetically closely related to *Dactylogyrus* spp. from Moroccan *Carasobarbus* (Torini), while the other Iberian *Dactylogyrus* lineage was more related to *Dactylogyrus* from Moroccan *Luciobarbus* and clustered with European species. Benovics et al. [2] subsequently found that *Dactylogyrus* species endemic to Iberian leuciscids are positioned in the same clade as European *Dactylogyrus* species, i.e., in the most diversified *Dactylogyrus* clade, suggesting rapid adaptive radiation of *Dactylogyrus* in this geographically isolated region, and multiple dispersion events of *Dactylogyrus* species in the Iberian Peninsula. Consequently, Benovics et al. [5], considering the Middle East as a historical geographic crossroads between Asia, Africa, and Europe, investigated the role of the Middle East in the diversification of *Dactylogyrus*. They inferred that the phylogeny of *Dactylogyrus* lineages follows the phylogeny of their associated cyprinid lineages and is interconnected with the historical dispersion of cyprinids in the peri-Mediterranean. They even found that the associations of individual *Dactylogyrus* lineages with the particular dispersal events proposed for cyprinids are also reflected in the morphological characters of the parasite attachment organ.

**Figure 3:**
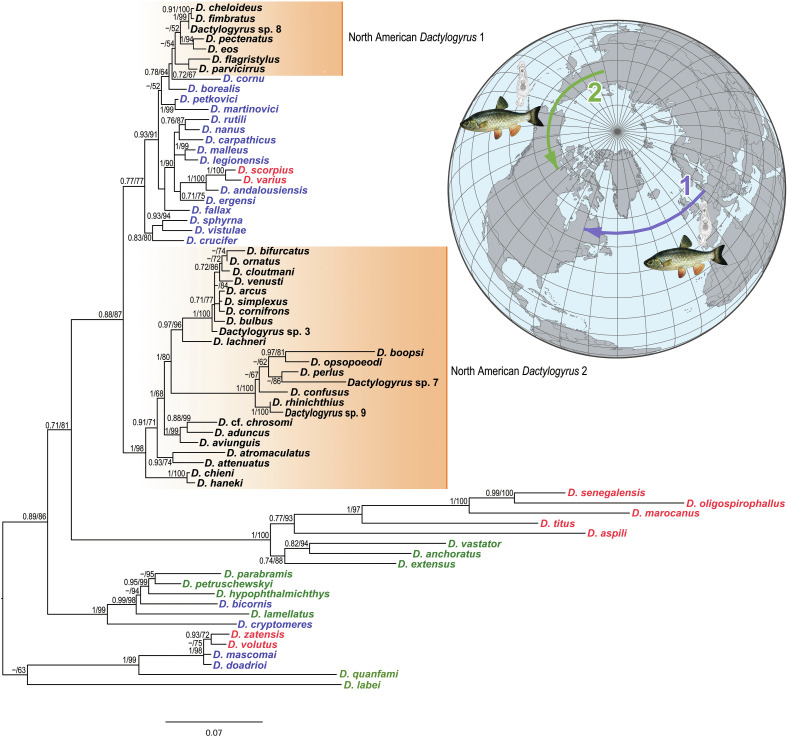
Scenario of historical biogeographical dispersals of Nearctic cyprinoids (leuciscids) inferred from the phylogenetic study of their *Dactylogyrus* spp. The position of the North American *Dactylogyrus* 1 lineage in the phylogenetic tree revealed the historical routes of leuciscid dispersion from West Eurasia to North America (in blue). The position of the North American *Dactylogyrus* 2 lineage in the phylogenetic tree suggests the historical biogeographical route of leuciscid dispersion from East Asia to North America (in green). The phylogenetic tree is modified from Šimková et al. [62]. *Dactylogyrus* spp. from fish species originated from different continents are shown by different colors in the phylogenetic tree as follows: *Dactylogyrus* spp. from Africa in red, *Dactylogyrus* spp. from Europe in bleu, *Dactylogyrus* spp. from Asia in green, and *Dactylogyrus* spp. from North America in black.

Finally, Šimková et al. [67] used *Dactylogyrus* parasites to investigate the historical dispersal of cyprinoids from West Eurasia and from East Eurasia to North America (Fig. 3). Concerning cyprinoids, only representatives of Leuciscidae are native in the Nearctic region. The study by Šimková et al. [67] revealed two Nearctic *Dactylogyrus* lineages, one of them restricted to the north-eastern parts of the United States and less diversified, this clade showing phylogenetic proximity to European *Dactylogyrus* fauna, suggesting the roles of the Thulean and De Geer land bridges in the historical dispersal of leuciscids from West Eurasia to North America. The Thulean Bridge is considered to be the most important route for the exchange of European and North American biota in the Early Tertiary. This land bridge connected southern Europe to eastern North America and was closed in the Early Eocene [30, 31]. The second trans-Atlantic connection responsible for European-North America biota exchange was the northern De Greer Bridge between Scandinavia and eastern North America, persisting until the Late Eocene. The second highly diversified Nearctic *Dactylogyrus* lineage revealed by Šimková et al. [67] included *Dactylogyrus* species only from Nearctic leuciscids, these fish collected in north-eastern and southern parts of the United States. An Asian (or East Eurasian) origin for this clade was proposed, likely associated with the colonization of North America by fish from Eastern Eurasia via the Beringia land bridge in the mid-Oligocene [11, 56].

## Genetic coadaptation limiting the presence of host-specific monogeneans in fish hybrids

The host-specificity of monogeneans suggests close genetic coadaptation between these parasites and their fish hosts, resulting from their reciprocal coevolutionary interactions. One of the possible ways to study the influence of host genetic background on the susceptibility of host-specific parasites is to conduct experiments involving pure genetic species (parental species) and their hybrids.

Hybridization is a common phenomenon documented in fish. Generally, hybrids of F1 generations are frequently characterized by hybrid vigor (hybrid heterosis or heterosis advantage), i.e., the hybrids exhibit superior vigor-related traits in comparison to their parental species. In contrast, the hybrids of post-F1 generations show reduced fitness, i.e., express hybrid breakdown, which results from genetic incompatibilities predicted by Dobzhansky [16] and Muller [35]. Thus, hybrids of post-F1 generations express many disadvantageous traits, often resulting from the disruption of gene expression regulation. However, the patterns of hybrid breakdown are more complex, as genetic incompatibilities correspond to the disruption of coadapted gene complexes in the organism. Such incompatibilities can arise due to different inheritance between the organelle genome (mostly maternal) and the nuclear genome (biparental) [9, 22, 45]. As a result, paternal (inter-mitotype) hybrid backcrosses have mismatched mitochondrial and nuclear genomes, a pattern restricted to paternal backcrosses, which is understood as the disruption of mito-nuclear (or cytonuclear) gene interactions, and is not present in maternal (intra-mitotype) hybrid backcrosses [9, 19].

Parasite load is considered an important measure of host vigor and may reflect either hybrid heterosis or hybrid breakdown [66, 68]. From this point of view, F1 hybrids express higher resistance to parasites, while post-F1 hybrids suffer from low immunity performance, and thus higher susceptibility to parasites (e.g., [27, 28, 64, 66]). Fritz et al. [20] proposed four static scenarios to explain the pattern of resistance and susceptibility to parasites in hybrids and parental species: (1) the additive scenario, predicting that resistance to parasites in hybrids is similar to the average resistance of the parental taxa, (2) the dominance scenario, predicting that resistance to parasites in hybrids is similar to that of one of the parental taxa, (3) the hybrid resistance scenario, predicting a higher resistance to parasites in hybrids when compared to both parental taxa, and (4) the hybrid susceptibility scenario, predicting higher susceptibility to parasites in hybrids when compared to parental taxa. However, Wolinska et al. [74] highlighted the role of negative frequency-dependent selection in host-parasite coevolution predicted by the Red Queen hypothesis, and proposed the existence of dynamic parasite infection in a hybridizing host system based on the frequencies of parental and hybrid genotypes.

Concerning monogenean parasites investigated in cyprinoid fish and their intergeneric F1 hybrids, the infection of F1 hybrids is lower when compared to parental species, which is in line with the hybrid heterosis hypothesis [13, 27, 28, 64]. This pattern of parasite infection in F1 hybrids is more pronounced for monogeneans specific to one or the other parental species than in monogeneans shared by both parental species [64]. The findings of low monogenean infection in F1 hybrids of various cyprinoid (both cyprinid and leuciscid) hybridizing systems studied in nature or in experiments are also compatible with the hybrid resistance scenario predicted by Fritz et al. [20], i.e., host-specific monogeneans reach high parasite load in associated hosts when compared to hybrid hosts, and this pattern is not affected by the frequency of host genotypes in experiments [13, 28].

Usually, each cyprinoid fish host species harbors at least some host-specific monogeneans. The coadaptation between host-specific monogeneans and associated fish hosts should preclude the presence of host-specific parasites on foreign host genotypes. The presence of almost all monogenean species specific to one or to the other parental species has been reported in intergeneric F1 hybrids of cyprinoid fish, indicating that there is not very strict coadaptation between host-specific monogeneans and associated host species [13, 27, 28, 64]. For example, Krasnovyd et al. [27] studied host-specific monogeneans in common bream (*Abramis brama*), roach (*Rutilus rutilus*), and their F1 hybrids from nature and reported 11 monogenean species (10 *Dactylogyrus* spp. and *Paradiplozoon homoion*) associated with roach and 5 monogenean species (3 *Dactylogyrus* spp., one *Gyrodactylus* species and *Diplozoon paradoxum*) associated with common bream, while F1 hybrids harbored 15 of these monogenean species. However, some limits given by host-parasite coadaptation may still preclude host-specific monogeneans from reaching high intensities of infection on hybrid hosts that are not genetically coadapted to host-specific monogeneans.

**Figure 4:**
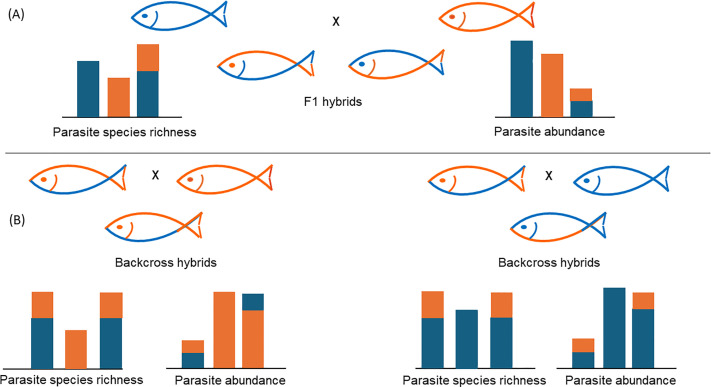
Patterns of parasite species richness and abundance observed in fish hybrids. (A) F1 hybrids result from the crossing of two genetically divergent parental species. Higher species richness but lower parasite abundance are observed in F1 hybrids when compared to parental species; (B) Backcross hybrids result from the crossing of F1 hybrids and one or the other parental species. Parasite species richness is higher in backcross hybrids when compared to the parental species involved in a crossing, but is not different when compared to F1 hybrids. Parasite abundance in backcross hybrids is similar to that in parental species involved in a crossing; however, backcross hybrids are more infected by host-specific parasites associated with a parental species involved in a crossing (orange in the left backcrossing scenario and blue in the right backcrossing scenario).

The susceptibility to monogenean infection in fish hybrids was verified also under experimental conditions using cyprinoid (leuciscid) species with higher genetic divergence (*A. brama* and *R. rutilus*) [13] and using cyprinoid (leuciscid) species with lower genetic divergence (silver bream (*Blicca bjoerkna*) and *A. brama*) [28], adjusting the experiments to achieve similar frequencies of two parental species and their F1 hybrids. Both studies using fish hybrid systems obtained by artificial breeding again showed that each of the parental cyprinoid species harbored host-specific monogenean fauna, while the hybrids harbored all monogenean species associated with one or the other parental species. Monogenean infection levels were still lower in hybrids (Fig. 4). In addition, both studies showed a very interesting pattern, i.e., an asymmetrical distribution of parental species-specific parasites in F1 hybrids – specifically, these hybrids were more infected by roach-specific monogenean parasites [27] or silver bream-specific monogenean parasites [28] than by common bream-specific monogenean parasites. Such an asymmetrical distribution of parental species-specific parasites in hybrids was interpreted as a potential result of the more limited inheritance of protective immunological mechanisms from one parental species than from the other. This may also indicate different degrees of coadaptation between different parental species and their host-specific parasites – in this case, *Dactylogyrus* (i.e. stronger coadaptation between common bream and its specific *Dactylogyrus* parasites than between roach and its specific *Dactylogyrus* parasites or between silver bream and its specific *Dactylogyrus* parasites).

Finally, Dedić et al. [13] investigated the effects of cytonuclear incompatibilities in backcross generations of hybrids using experimental infection by monogeneans. They suggested that cytonuclear incompatibilities do not play a significant role in determining the load of monogeneans specific to one or the other parental species. Specifically, they showed similar levels of monogenean infection in backcross generations of hybrids and pure (parental) species (Fig. 4B). The presence of different asymmetrical distributions of parental taxa-associated parasites in the two backcross generations was reported (Fig. 4B); however, this was more consistent with host-parasite coadaptation than with hybrid breakdown. Thus, backcross hybrids with a higher proportion of the genes of one parental taxon also exhibited a high level of this parent’s taxon-associated parasites. Therefore, host-parasite coevolutionary interactions seem to play an obvious role in determining the level of infection of host-specific monogeneans in hybrids of post-F1 generations.

## Conclusions

Research focused on congeneric monogeneans has revealed their high species diversity and morphological variability. Living on the same host is a common pattern in congeneric monogeneans, this coexistence is facilitated by morphological adaptation, host and microhabitat specificity, and reproductive isolation. In spite of the high host specificity observed in congeneric monogeneans, their speciation and diversification have been inferred to be mostly associated with the host switch facilitated by the sympatric occurrence of their phylogenetically related hosts. Additional coevolutionary events have been documented to play a role in the speciation of congeneric monogeneans; the significance of intrahost speciation and cospeciation in several fish-monogenean systems was highlighted. Congeneric monogeneans represent a useful tool to investigate the historical biogeography of their freshwater fish hosts, a group with a currently fragmented distribution. Host-specific monogeneans are associated with their own hosts; however, their presence in hybrid hosts is not restricted by host-parasite coadaptation, even if coadaptation plays a role in limiting the load of specific parasites in intergeneric fish hybrids.

## Glossary of key terms

**Basic host specificity** – number of host species infected by a given parasite species.

**Phylogenetic host specificity** – estimate of host specificity considering phylogeny of host species parasitized by a given parasite species.

**Cospeciation** – parasite speciation in response to the speciation of a host.

**Host switch** – parasite colonization of a new host species followed by speciation. Host switching is promoted in the case of hosts living in sympatry.

**Intra-host speciation** (parasite duplication) – parasite speciation within a host species, which leads to two or more lineages of parasites present on the same host species.

**Sorting events** – (1) when a parasite became extinct from a host lineage after a cospeciation event (extinction), or (2) when a parasite was absent from the host founder population at a host speciation event (“missed the boat”).

**Failure to diverge** – when a parasite failed to diverge during the speciation process of its host and therefore, the same parasite species is present on two recent, phylogenetically close host lineages.

**Disjunctive distribution** – separation of populations from each other geographically. This occurs when the habitat of a species is fragmented, which produces fragmented populations, and when that fragmentation becomes so divergent that species dispersal between one suitable habitat to the next is disrupted, and isolated population can be produced.

**Host-parasite coadaptation** – process by which the host and parasite undergo reciprocal and mutual adaptations. Host-parasite coadaptation is often seen as evidence for coevolution.

**F1 hybrids** – the first filial generation of offspring of distinctly different parental types (or species). They often exhibit hybrid vigor, i.e., enhanced longevity and immunity to diseases.

**Backcross hybrids** – the generation of hybrids resulting from the crossing of a heterozygote (individuals of the F1 generation) with individuals of the parental generation.

**Genetic coadaptation** – mutual adaptations of the genes. A gene may be favored by selection if it is in the same individual as a particular gene at another locus. Genetic coadaptation may be broken by hybridization. Hybridization between species with differently coadapted gene or chromosomal complexes may result in a decrease in fertility, viability, and survival, especially in post-F1 generations of hybrids.

**Mito-nuclear (or cytonuclear) coadaptation** – coadaptation required for proper cell function. The selection for optimal mito-nuclear interactions results in coadaptation between mitochondrial and nuclear genomes. Mito-nuclear coadaptation may be broken by hybridization (mostly documented in paternal backcross hybrids).
